# Change in Ratio of Observed-to-Expected Deaths in Pediatric Patients after Implementing a Closed Policy in an Adult ICU That Admits Children

**DOI:** 10.1155/2012/674262

**Published:** 2012-05-08

**Authors:** Yoshitoyo Ueno, Hideaki Imanaka, Jun Oto, Masaji Nishimura

**Affiliations:** ^1^The University of Tokushima Graduate School, Tokushima, Japan; ^2^Department of Emergency and Disaster Medicine, Tokushima University Hospital, 3-18-15 Kuramoto Tokushima 770-8503, Tokushima, Japan; ^3^Department of Emergency and Critical Care Medicine, Tokushima University Hospital, Tokushima, Japan

## Abstract

*Backgrounds*. We examined the effect on the prognosis of critically ill pediatric patients after a closed ICU policy was implemented into an adult ICU that admitted children. *Materials and Methods*. We assessed the Pediatric Index of Mortality 2 (PIM2) score of pediatric patients (≤15 y.o.) admitted to the ICU from 2001 to 2009. In our teaching hospital, the department for intensive care was established in January 2004. Since then, for critical care patients, we have followed a closed ICU policy with full-time intensivists. We subsequently compared PIM2 scores and the ratio of observed-to-expected deaths (O/E ratio) for three three-year periods: 2001–2003 (before closed policy), 2004–2006, and 2007–2009. *Results*. Data was collected from 532 pediatric patients. While the PIM2 score statistically significantly increased from 0.066 ± 0.130 for 2001–2003 to 0.114 ± 0.239 for 2004–2006 and 0.086 ± 0.147 for 2007–2009, the O/E ratio decreased from 1.49 for 2001–2003 to 0.82 for 2004–2006 and remained at 0.82 for 2007–2009. *Conclusion*. The O/E ratio for critically ill pediatric patients improved after the establishment of a closed policy in an adult ICU that admitted children.

## 1. Introduction

There are two main kinds of ICU policy: closed and open. In a closed ICU, full-time intensivists provide patient care. In open ICUs, patient care is provided by primary physicians. Evidence from a number of studies suggests that the prognosis of critically ill patients is improved in closed ICUs that provide 24-hour care by intensivists [1–3]. While it has been reported that greater input by intensivists results in more efficient resource use and leads to better outcomes for critically ills patients, evidence has not yet been presented specifically for pediatric patients.

Most pediatric ICUs in the United States are staffed by full-time intensivists in a “closed” ICU model. However, in Japan as well as in many Asian countries, critically ill children are cared for in adult ICUs because of a deficiency of pediatric ICU beds. Therefore, it is meaningful to investigate the outcomes in children admitted to adult ICUs.

Originally in our adult acute-care ICU, each department admitted patients under their service. The ICU staff mainly managed bed control and participated in patient care only when they were consulted on, otherwise known as “open” ICU model. When the ICU was reestablished in January 2004 as a department of emergency and critical care medicine, we have employed a closed policy with 24-hour care provided by full-time intensivists who do daily rounds and decide mechanical ventilator settings, cardiovascular treatment, and administration of antimicrobial agents. The whole staff in our ICU have meeting and round twice a day, discussing patient problem and care there. We conducted this study to investigate whether the introduction of a closed ICU policy into an adult ICU improved the prognosis of the critically ill pediatric patients.

## 2. Patients and Methods

We obtained data retrospectively for critically ill pediatric patients (≤15 y.o.) admitted to our ICU, which accepted infants to adults in the nine years from January 2001 to December 2009. To adjust for the severity of illness of these pediatric patients, we assessed Pediatric Index of Mortality 2 (PIM2) scores and calculated the ratio of observed-to-expected deaths (O/E ratio). PIM2 was assessed from records made during the first hour of ICU admission for systolic blood pressure, pupillary reaction to bright light, PaO_2_ and F_I_O_2_, base excess, mechanical ventilation, main reason for ICU admission, admission following cardiac bypass, and risk of diagnosis [4, 5]. The PIM2 score does not predict risk for individual patients but, rather, indicates overall risk of mortality and has been proposed as a useful means for continuously monitoring the quality of pediatric intensive care. To ensure adequate population size, we decided to include all patients in the three-year period before the closed policy was implemented. Then, to test for confounding variables, we also compared data for patients admitted during two three-year periods after the closed policy was implemented. Consequently, we compared group data for critically ill pediatric patients in the following periods: 2001–2003, 2004–2006, and 2007–2009. By summing the PIM2 scores for every patient, we calculated the expected number of deaths and then calculated O/E ratios by dividing the observed number of deaths by the expected number of deaths in each period. As a standard for assessing the clinical performance of ICUs [6], the O/E ratio has been used to classify the levels of performance of different ICUs [7]. We also calculated PIM2 scores and O/E ratios for subgroups of patients with and without congenital heart anomalies. Differences were considered significant when *P* < 0.05. All statistical analysis was performed with statistical software (SPSS 11.01, SPSS, Chicago, IL).

## 3. Results

532 pediatric patients admitted to our ICU during 2001 to 2009: median (25% to 75% quartiles) for age was 1 year old (5 months old to 6 years old), for body weight was 8.6 kg (4.1 to 15.8 kg), and for height was 74 cm (54 to 106 cm). The number was 12.6% of all admissions (4,208 patients). We found congenital heart anomalies in about half of the patients (Table 1). The PIM2 score increased from 0.066 ± 0.130 for 2001–2003 to 0.114 ± 0.239 for 2004–2006 and 0.086 ± 0.147 for 2007–2009 (Table 2). Table 2 shows the number of expected deaths and observed deaths. The O/E ratio decreased from 1.49 for 2001–2003 to 0.82 for 2004–2006 and remained at 0.82 for 2007–2009. The O/E ratio decreased for patients without congenital heart anomalies (3.52 for 2001–2003, 0.78 for 2004–2006 and 0.69 for 2007–2009), but did not change for patients with congenital heart anomalies.  Table 3 shows mortality according to PIM2 score. Mortality for patients with PIM2 score <5% decreased from 6.8% for 2001–2003 to 0.8% for 2004–2006 and 0% for 2007–2009. Mortality for patients with PIM2 score >5% was not different among the three periods.

## 4. Discussion

The main findings of this study are (1) after the closed ICU policy was implemented into an adult ICU, the O/E ratio for critically ill pediatric patients improved and (2) the improvement in prognosis was better for low risk patients than for high-risk patients.

While closed ICUs have been shown to improve the prognosis of critically ill adult patients, from the literature, we could not confirm whether this was also true for pediatric patients. Although our findings derive from a retrospective study based on historical observation, as far as we know, this is the first study to report how a closed policy affects outcome for critically ill pediatric patients in an adult ICU.

We assumed that a closed ICU policy would decrease preventable deaths in low-risk patients: we found that the mortality of patients with PIM2 score <5% decreased from 6.8% to 0.8% and 0% (Table 3), which suggests that fewer mild cases died after the closed ICU policy was implemented. While we did not investigate the cause of death in each patient, this lower mortality most probably resulted from the occurrence of fewer serious complications and more rapid containment of life-threatening trouble after the closed ICU policy was implemented. Since the majority of patients in this study were low-risk (PIM2 score <5%), this decrease in such preventable deaths was even more worthwhile.

Our findings suggest that while the closed ICU policy decreased mortality for patients without cardiac anomalies, it did not do so for patients with cardiac anomalies. This discrepancy may be due to a problem in the PIM2 scoring system: Czaja et al. have reported that PIM2 scoring does not adequately adjust risk for cardiac surgery [5]. 

This study has a number of limitations. First, because it is based on a retrospective survey that covers a long period, factors other than the change in ICU policy may have contributed to the improvement. Against this, we did find that the O/E ratio was identical in the periods 2004–2006 and 2007–2009, which is good supporting evidence that the change in ICU policy was mainly responsible. We tried to minimize selection bias by obtaining the data from computerized charts but we could not neglect the possibility of such bias. In addition, we did not have detailed information regarding respiratory condition, the origin of shock, or failed organs. Second, because the outcome for pediatric patients managed in pediatric ICUs is reportedly better than that in general ICUs [8–11], perhaps a before-and-after comparison of a PICU would be more relevant; indeed, we would prefer that all critically ill pediatric patients were managed in specific units. In Japan, however, PICU beds are in short supply. Consequently, the majority of critically ill pediatrics receive care in adult ICUs. In our less-than-ideal world, this study has some practical relevance. Third, predictive models, including PIM2, are affected by many factors [12, 13]. We chose the PIM2 scoring system, because it requires data only from the first hour after admission and reasons for admission [10, 14]. Using O/E ratio, we adjusted for the severity of illness and compared predicted with actual mortality. The validity of the O/E ratio has been supported by Rapoport et al., who suggested that the O/E ratio may be useful for classifying levels of ICU performance [7]. At the same time, other mortality prediction models also have limitations: the PRISM (Pediatric Risk of Mortality) scoring system, possibly better than PIM2 system, requires a longer period of evaluation (12 or 24 hrs), and in SAPS (Simplified Acute Physiology Score), mortality tends to be overestimated for surgical patients and underestimated for medical patients. Fourth, raw mortality in our ICU was relatively high: 7.0 to 9.7%. The reason of high mortality was not clear but we speculated it might be because very sick children were admitted to the ICU due to limited number of ICU beds (10 ICU beds for 696 hospital beds).

In conclusion, the O/E ratio in critically ill pediatric patients improved after the establishment of a closed policy in a general ICU.

## Figures and Tables

**Table 1:** Patient profile.

	2001–2003 (*N* = 194)	2004–2006 (*N* = 181)	2007–2009 (*N* = 157)
Male	90 (46%)	94 (52%)	67 (43%)
Age (y.o.)	2.8 ± 3.7	3.5 ± 3.9	4.1 ± 4.6
Mechanical ventilation	155	177	137
Background diseases			
Congenital heart anomalies	106	101	96
Neurosurgical diseases	17	16	12
Respiratory diseases	20	15	11
Hematologic diseases	6	9	0
Neurologic diseases	11	4	1
Trauma	7	2	3
Cardiopulmonary arrest	9	9	4
Others	18	25	30

**Table 2:** PIM2 score and observed deaths.

	2001–2003	2004–2006	2007–2009
PIM2 score	0.066 ± 0.130	0.114 ± 0.239	0.086 ± 0.147
Observed deaths	19	17	11
Expected deaths	12.75	20.71	13.43
O/E ratio	1.49	0.82	0.82

**Table 3:** Mortality according to PIM2 score.

	2001–2003	2004–2006	2007–2009
PIM2 score			
<5%	6.8% (*N* = 147)	0.8% (*N* = 123)	0% (*N* = 96)
5–20%	15.6% (*N* = 32)	15.0% (*N* = 40)	11.4% (*N* = 44)
>20%	26.7% (*N* = 15)	55.6% (*N* = 18)	35.3% (*N* = 17)

## Abbreviation

ICU: Intensive care unit
O/E ratio: Observed/expected deaths ratio
PIM2: Pediatric Index of Mortality 2.
